# Comparison of Non-Invasive and Minimally Invasive Preimplantation Genetic Testing for Aneuploidy Using Samples Derived from the Same Embryo Culture

**DOI:** 10.3390/jcm14010033

**Published:** 2024-12-25

**Authors:** Anna Bednarska-Czerwińska, Joanna Smoleń-Dzirba, Anna Strychalska, Wojciech Sierka, Urszula Wróblewska, Patrycja Mermer, Barbara Masarczyk, Natalia Jodłowiec-Lubańska, Anna Kokot, Klaudia Simka-Lampa, Nikola Zmarzły, Emilia Morawiec, Aneta Orczyk, Beniamin Oskar Grabarek

**Affiliations:** 1 Laboratory of Molecular Biology and Virology, Gyncentrum, 40-851 Katowice, Poland; j.smolen-dzirba@gyncentrum.pl (J.S.-D.); a.strychalska@gyncentrum.pl (A.S.); w.sierka@gyncentrum.pl (W.S.); u.wroblewska@gyncentrum.pl (U.W.); p.mermer@gyncentrum.pl (P.M.); b.masarczyk@gyncentrum.pl (B.M.); n.jodlowiec-lubanska@gyncentrum.pl (N.J.-L.); a.kokot@gyncentrum.pl (A.K.); simka.klaudia@gmail.com (K.S.-L.); e.morawiec@gyncentrum.pl (E.M.); 2 Collegium Medicum, WSB University, 41-300 Dąbrowa Górnicza, Poland; nikola.zmarzly@wsb.edu.pl (N.Z.); aorczyk@wsb.edu.pl (A.O.); bgrabarek7@gmail.com (B.O.G.); 3 Department of Microbiology, Faculty of Medicine, Academy of Silesia, 41-800 Zabrze, Poland

**Keywords:** non-invasive/minimally invasive preimplantation genetic testing for aneuploidy, spent culture medium, blastocoel fluid, embryo transfer

## Abstract

**Background/Objectives**: To assess the ploidy status of embryos via preimplantation genetic testing for aneuploidy (PGT-A), a biopsy of trophectoderm (TE) cells can be performed. However, this approach is considered invasive, and therefore the aim of this study was to identify the optimal sample type and sampling day for non-invasive or minimally invasive PGT-A (ni/miPGT-A) in terms of data quality and concordance rates with TE biopsies derived from the same embryos. **Methods**: This study was performed using 239 embryo cultures. After optimization using 96 embryos, non-invasive spent culture media (SCM) and a minimally invasive combination of blastocoel fluid and SCM (BF+SCM), along with the corresponding TE samples, were collected from 143 embryos cultured for 5 days (n = 70) or 6 days (n = 73), and all were subjected to ni/miPGT-A with whole-genome amplification followed by next-generation sequencing. **Results**: The amplification failure rate was lower for SCM samples than for BF+SCM (SCM: 0.7%, 1/143 vs. BF+SCM: 7.7%, 11/143; *p* = 0.005). The rate of ploidy concordance with TE was significantly higher for SCM samples than for BF+SCM samples (SCM: 83.7%, 118/141 vs. BF+SCM: 58%, 76/131; *p* < 0.001). Among SCM samples, concordance rates were higher for samples derived from embryos cultured for 6 days (87.5%, 63/72) than for 5 days (79.7%, 55/69). In the embryos cultured for 6 days, discordant cases included five (6.9%) SCM samples with falsely negative (euploid) results that were deemed to be mosaic according to TE and four (5.6%) samples falsely found to be aneuploid. **Conclusions**: SCM samples derived from embryos cultured for 6 days can be applied in niPGT-A with subsequent verification of aneuploid samples using TE biopsy.

## 1. Introduction

Preimplantation genetic testing for aneuploidy (PGT-A) is a tool used in assisted reproduction technology (ART) practices to identify embryos with numerical chromosomal aberrations and distinguish them from euploid embryos, which are considered to have high developmental potential and are thus suitable for uterine transfer. The objectives of performing PGT-A analyses within in vitro fertilization (IVF) procedures include (i) increasing pregnancy rates per embryo transfer, (ii) decreasing miscarriage rates, (iii) increasing elective single embryo transfer, and (iv) reducing time to achieve pregnancy [1]. To assess the ploidy status of embryos with traditional PGT-A tests, invasive methods of sampling need to be applied. These invasive sampling methods include the removal of both polar bodies from oocytes/zygotes, the biopsy of 1–2 blastomeres from cleavage-stage embryos (day 3 post fertilization), or the biopsy of 5–10 trophectoderm (TE) cells from blastocyst-stage embryos after 5–6 days of culture. While the first two methods are currently rarely used, trophectoderm biopsy is still widely performed, and along with subsequent DNA isolation, whole-genome amplification (WGA), and analysis by next-generation sequencing (NGS), it is currently the method of choice and the most common approach to preimplantation screening for aneuploidy [2,3,4,5].

Although biopsy of trophectoderm cells for the purpose of PGT-A is now routinely performed in embryology labs and appears to be safe for embryos [6], it has been shown that the removal of larger numbers of TE cells during blastocyst biopsy may adversely affect implantation rates, leading to unsatisfactory outcomes of IVF procedures following PGT-A [7]. In addition to a presumably reduced implantation potential, major concerns with such invasive PGT-A approaches include unknown long-term impacts of embryo biopsy on the offspring and increased risk of maternal hypertensive disorders [8,9,10]. Another difficulty arising in TE testing for aneuploidy is related to the possibility of detecting mosaic chromosome profiles, i.e., NGS profiles with an intermediate chromosome copy number (for instance between 1 and 2 (mosaic monosomy) or between 2 and 3 (mosaic trisomy)), as the proper interpretation and transfer decision making for such embryos remain uncertain [11]. Furthermore, it is essential that biopsies are performed by well-trained and highly experienced embryologists, which generates additional costs for assisted reproductive procedures [5,12]. Therefore, it has been proposed to omit the embryo biopsy step and use less invasive techniques aimed at obtaining DNA representative for entire embryos and concurrently suitable for application in the genetic testing procedures for aneuploidy [12,13].

Materials such as spent culture medium (SCM), blastocoel fluid (BF), and a mixture of both fluids (BF+SCM) were found to contain cell-free DNA, and thus were considered as potential options for use in non-invasive (with SCM collection) or minimally invasive (with collection of BF or BF+SCM) variants of preimplantation genetic testing [14,15,16]. In several studies, the applicability of these three types of samples in preimplantation genetic testing was investigated, with results varying between studies in terms of the rate of amplification failure and concordance with the reference sample obtained from a corresponding embryo (i.e., TE biopsy, inner cell mass, whole blastocyst) [12,13,16,17,18,19,20,21,22,23,24,25,26,27,28,29,30,31,32,33,34]. Discrepancies between the reported results were evident even after stratification by the examined non-invasive/minimally invasive sample type. It should also be noted that most of these studies were focused on the evaluation of amplification failure and concordance rates for only one type of non-invasive [12,13,17,19,20,21,22,23,24,25,26] or one type of minimally invasive sample [16,27,28,29,30,31,32,33,34]. Given that the discrepancies between the results reported in previous studies can, at least in part, be explained by (i) different culturing methodologies, (ii) manipulations applied to embryos (such as assisted hatching, vitrification, and devitrification), as well as (iii) different approaches to DNA analysis (i.e., different amplification and sequencing methods [12,13,16,17,18,19,20,21,22,23,24,25,26,27,28,29,30,31,32,33,34]), it is difficult to perform reliable comparisons of the effectiveness of using different types of non-invasive/minimally invasive samples. Additionally, when transferring the research results to clinical practice, modifications to standard embryo culture protocols should be considered in order to prevent maternal DNA contamination of the samples collected in a non-invasive or minimally invasive manner, as well as to improve the quantity of cfDNA in these samples, which should reduce amplification failure rates and enhance concordance rates with TE biopsy-based PGT-A. Still, validation experiments are needed before ni/miPGT-A can be accepted for clinical use [35,36,37].

The aim of this study was to determine which of two non-invasive/minimally invasive sample types, i.e., SCM or BF+SCM, both obtained from the same set of embryo cultures, would be the most suitable source of cell-free DNA for performing preimplantation genetic testing for aneuploidy in a clinical setting. Using the same embryos for collection of two types of non-invasive/minimally invasive samples along with a TE biopsy and subjecting all of these samples to the same WGA and NGS PGT-A methods provides an opportunity to obtain results that can be reliably compared, and thus used to decide on their possible routine application. Additionally, we intended to determine whether the time of embryo culture (5 vs. 6 days) affects the performance or the results of non-invasive preimplantation genetic tests. The most suitable material in terms of sample type and culture duration for assessing chromosomal status of the embryo without the need for a TE biopsy was selected based on (i) the lowest whole-genome amplification failure rates and (ii) the highest concordance between results obtained for non-invasive/minimally invasive sample types with the result of traditional invasive PGT-A performed with trophectoderm cells. Additionally, we attempted to compare non-invasive samples that were concordant with the corresponding TE biopsy with those that were discordant.

## 2. Materials and Methods

This study was conducted according to the guidelines of the Declaration of Helsinki and approved by the Institution of the Bioethical Committee operating at the Regional Medical Chamber in Krakow (No. 161/KBL/OIL/2021). Informed consent for participation in the study and publication of this article was obtained from all patients.

### 2.1. Study Group and Design

**Figure 1:**
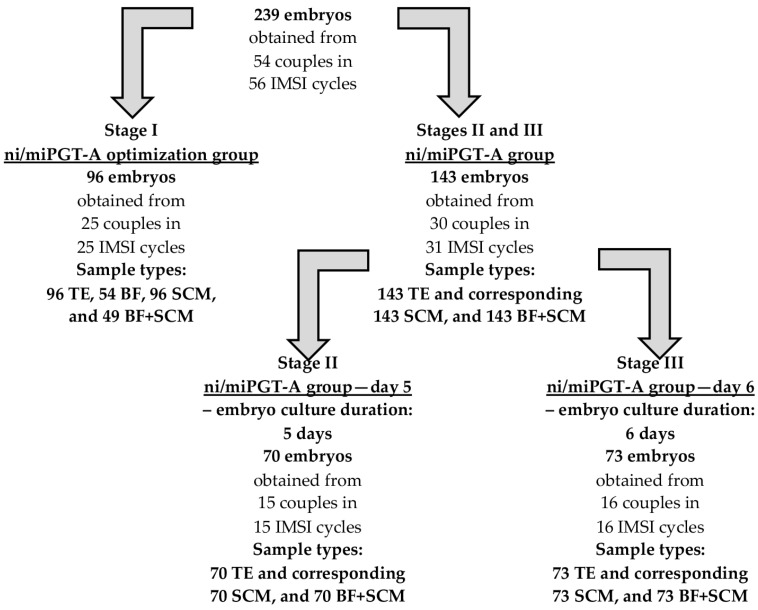
Study groups and sample types tested at each stage of the study. IMSI—intracytoplasmic morphologically selected sperm injection; ni/miPGT-A—non-invasive/minimally invasive preimplantation genetic testing for aneuploidy; TE—trophectoderm cells; BF—blastocoel fluid; SCM—spent culture medium.

Samples obtained from embryo cultures conducted for 54 couples, who agreed to participate in the project, were examined in this study. All patients were of Caucasian origin, and their characteristics are shown in Table S1. All couples visited the private IVF center, Gyncentrum Sp. z o. o., based in Katowice, Poland, between January 2021 and July 2022, and all were qualified for infertility treatment with IVF using the IMSI (Intracytoplasmic Morphologically Selected Sperm Injection) procedure. During the study period, all couples underwent at least one IMSI cycle, and two couples participated in two cycles. All the couples had their embryos subjected to TE biopsy in order to perform conventional PGT-A, and the main reason for the testing was the advanced maternal age—35 out of 54 (64.8%) of women were 35 years old or older. The median age of the female partner was 36 years, the youngest woman was 26 years old, and the oldest was 43. Couples with severe male factor constituted about 20% (11/54) of the group, and the median age of the male partners was 37 years, with the youngest man being 28 years old and the oldest being 60. More than half of the group (51.9%, 28/54) were patients with previous unsuccessful IVF history. This category includes couples with up to 2 implantation failures, as well as those who have undergone IVF procedures but failed to obtain embryos suitable for transfer. In six couples (11.1%), one partner had an abnormal karyotype, which was the reason for the preimplantation genetic testing. In the studied group, there were 461 MII oocytes eligible for fertilization by IMSI procedure, and 276 viable embryos were obtained, of which 239 were subjected to TE-based PGT-A analyses along with non-invasive/minimally invasive PGT-A trial (with collection of SCM, BF, BF+SCM), collectively referred to here as ni/miPGT-A (Table S1). Not all obtained viable embryos were used in the study, as seven couples who agreed to participate in the study were not willing to biopsy all of their embryos. For these patients, the selection of embryos for the study was based on the degree of development of the blastocoel cavity and a clearly visible inner cell mass (ICM) to ensure the safety of the embryonic node. During the study, trophectoderm cells and corresponding non-invasive and minimally invasive culture materials from 239 embryos were collected in a three-stage procedure (Figure 1).

First, 96 embryo cultures from 25 couples, which formed the “ni/miPGT-A optimization group”, were used in an initial, optimization phase of the study. In brief, for all 96 embryos obtained within this group, at least one of the minimally invasive or non-invasive materials, such as blastocoel fluid (BF, N = 54), spent culture medium (SCM, N = 96), or a mixture of both fluids (BF+SCM, N = 49) in addition to trophectoderm (TE, N = 96) cells, was collected. These fluids were used in the optimization of whole-genome amplification conditions. Due to the high rate of amplification failures observed for blastocoel fluid (39/54, (72.2%)), this minimally invasive material was not collected in further stages of the study.

Next, 143 embryo cultures from 30 couples formed the “ni/miPGT-A group” (one couple participated in 2 IMSI cycles and was incorporated to the study in both the ni/miPGT-A optimization group and the ni/miPGT-A group). All 143 embryos were subjected to TE biopsy along with the collection of SCM and a mixture of BF+SCM. All three materials (TE, SCM, and BF+SCM) were used for whole-genome amplification and were further tested for aneuploidy with the same next-generation sequencing technique. Comparison of patient characteristics showed no major differences between couples included in the ni/miPGT-A optimization group and the ni/miPGT-A group. The only statistically significant difference observed between groups was the lower frequency of viable embryos obtained in the optimization group (51% vs. 66.8%, *p* = 0.001) (Table S1).

Among the ni/miPGT-A group, aneuploidy testing was performed in two subgroups. Initially, analyses were performed for 70 embryos (from 15 couples) intentionally cultured until day 5 (“ni/miPGT-A group—day 5”). Afterwards, the same analyses were completed for 73 embryos (from 16 couples) purposefully cultured until day 6 (“ni/miPGT-A group—day 6”) to investigate the possible influence of the additional culture day on the ni/miPGT-A outcome. The sum of couples in both subgroups is greater than the total number of couples included in the ni/miPGT-A group, because one couple participated in 2 IMSI cycles and was included in both groups, i.e., the groups with 5 and 6 days of embryo culture. A comparison of couples and embryos between groups with different culturing durations is given in Table 1.

All couples included in the study underwent an IMSI cycle with their own fresh oocytes, and all but one used fresh sperm ejaculated on the day of the patient’s oocytes pickup. The only couple that used frozen donor sperm was included in the ni/miPGT-A optimization group (3 embryos obtained).

### 2.2. Embryo Cultures and Sample Collection

Ovarian stimulation was performed using standard gonadotropin-releasing hormone (GnRH) agonist, follicle-stimulating hormone protocol, or a combination of GnRH antagonist and follicle-stimulating hormone protocol. Oocytes were retrieved under the guidance of transvaginal ultrasound 36 h post injection of human chorionic gonadotropin. Cumulus oocyte complexes (COCs) obtained using the oocyte retrieval procedure were preincubated for 3 h (in a gas phase of 5% O_2_, 6% CO_2_, 89% N_2_ at 37 °C), followed by denuding of COCs cells in hyaluronidase enzyme. After removal of cumulus cells and rinsing of oocytes, all granular cells were thoroughly removed to avoid residual maternal contamination in biopsy samples. Oocytes in metaphase II (MII) were used for IMSI with standard IVF protocols and then cultured in a time-lapse incubator. Fertilization was confirmed 16–18 h post IMSI, after which blastocysts developed over a maximum 6-day incubation period in culture medium covered with mineral oil

Routine IVF culture conditions were applied until collection of non-invasive/minimally invasive samples and TE biopsy. The most adequate culture conditions, strategies, and media were used in a “closed” culture system to limit the exposure of embryos to suboptimal conditions and to help decide on the optimal time for the biopsy. Multi-well dishes and (micro)droplets in separate dishes were used for individual embryo culture to prevent mixing of the embryos due to accidental movement during handling.

All obtained prezygotes, zygotes, and embryos were cultured until day 3 in continuous media (SAGE 1-Step, CooperSurgical, Trumbull, CT, USA) in CultureCoin Embryo Culture Dish for MIRI TL, covered with a protective layer of oil (GM501 Mineral Oil, Gynemed, Lensahn, Germany). The dishes were pre-warmed in a CO_2_ incubator before being filled with pre-equilibrated medium. All culture wells with approximately 25 µL of pre-equilibrated IVF culture medium were handled within the workstation. All embryos in the culture wells were placed individually in a separate well (with 1 microdrop) and were set very precisely in the center of the well. On the fourth day of culture, the medium of each embryo was refreshed, which was preceded by washing the embryos in a series of three drops of fresh medium, each of 100 µL, using a new micropipette for each drop, and then embryo culture was continued in 10 µL of fresh medium until day five or six, when samples were collected.

The biopsy equipment set-up included an inverted microscope with a heated stage and three-dimensional micromanipulators and microinjectors (air or oil), placed on anti-vibration pads, corresponding to the set-up for IMSI procedures. In addition, a stereoscope (for transferring oocytes/embryos in biopsy dishes and for tubing) and incubators were available adjacent to the working area. The Octax laser (Vitrolife, Goteborg, Sweden) was used for the blastocyst biopsy.

A biopsy of five TE cells was performed for blastocyst-stage embryos (Gardner: B4), always with a clearly visible center of the blastocoel and the TE at the periphery of the embryo. TE sampling was routinely preceded by assisted hatching (AH) with a single laser pulse (Octax Laser) to open the zona pellucida (ZP). After installing the holding pipette and biopsy pipette with an internal tip diameter of 25 μm in the injection holder, blastocysts were transferred to the biopsy plate. Biopsies were performed in the buffered “biopsy medium” GM501 Wash (Gynemed, Lensahn, Germany), a ready-to-use medium for human embryo washing procedures and any short-term out-of-incubator procedures, as recommended by the manufacturer. Blastocysts were “fixed” at the 9 o’clock position with a holding pipette, with the ICM of the blastocyst placed between 7 and 11 o’clock, so that during TE biopsies it was clearly visible, distant from the ZP opening, and protected from suction using a holding pipette. The TE cells were aspirated into a biopsy pipette with gentle suction. Laser pulses were directed at cell-to-cell junctions to dissect aspirated cells from the blastocyst, or to minimize cell damage during mechanical detachment of TE cells by a quick flicking movement of the biopsy pipette against the holding pipette. To avoid cross-contamination during biopsy, the biopsy pipette was changed for each blastocyst.

After biopsy, blastocysts were immediately transferred to individualized droplets of culture medium in separate dishes until vitrification (Vit Kit Freeze, Irvine Scientific, Irvine, CA, USA). Biopsied TE cells were washed in phosphate-buffered saline (PBS) at least twice using a sterile transfer pipette before being transferred to the RNase- and DNase-free PCR reaction tubes with 1.5 μL of PBS. Special precautions were taken to avoid loss of genetic material between washing steps.

Non-invasive/minimally invasive sample types corresponding to each TE sample were collected prior to TE biopsy. A total of 5 μL of SCM was collected for each embryo directly from the CultureCoin Embryo Culture Dish, shortly after blastocyst transfer to the biopsy plate. All SCM samples were transferred to the RNase- and DNase-free PCR tubes using sterile filtered pipette tips. Sampling of BF and BF+SCM was performed on a biopsy plate. To collect BF from the cavity of expanded blastocysts, an intracytoplasmic sperm injection (ICSI) micropipette was gently inserted through the zona pellucida between the TE cells, and BF was aspirated and placed in the RNase- and DNase-free PCR reaction tubes with 1 μL of PBS. A single aspiration was performed to minimize possible cell damage and the risk of BF contamination by TE cells. The mixture of BF and SCM was collected after ICSI needle injection and blastocyst collapse with the release of blastocoel fluid to the medium; <5 μL of biopsy medium enriched with blastocoel fluid was combined with a ~5 μL SCM sample, and the BF+SCM mix was collected in RNase- and DNase-free PCR tubes using sterile filtered pipette tips. After all non-invasive and minimally invasive samples were collected, an opening in the zona pellucida far from the ICM was created during assisted hatching preceding TE biopsy. All samples were immediately frozen and stored at −80 °C until molecular testing.

### 2.3. Molecular Analysis

Whole-genome amplification (WGA) was performed within 2 weeks of sample collection, with the use of the SurePlex kit (SurePlex DNA Amplification System, Illumina, San Diego, CA, USA). The TE samples were amplified strictly following to the manufacturer’s instruction; processing BF, SCM, and BF+SCM samples in the optimization part of the study was performed with modifications to the original protocol, namely skipping the step of enzymatic lysis and increasing the number of cycles in pre-amplification and/or amplification steps. After each modification, amplification failure rates were controlled, and the best conditions were selected to be used in the next stages of the study. Due to the poor amplification of BF samples, observed irrespective of the modifications applied to the original protocol, this sample type was rejected from the next steps of the study. Finally, non-invasive/minimally invasive SCM and BF+SCM samples (~2.5 µL) collected at stages II and III of the study (Figure 1) were amplified according to the original SurePlex protocol, with a single modification that involved increasing the number of cycles in the amplification step from 14 to 20.

WGA products obtained at stages II and III of the study from TE cells and corresponding SCM and BF+SCM samples were visualized by agarose gel electrophoresis, quantified with the Quantus™ fluorometer using a QuantiFluor^®^ ONE dsDNA Dye System (Promega, Fitchburg, WI, USA), and subjected to the next-generation sequencing DNA library preparation using a VeriSeq PGS-MiSeq Kit (Illumina, San Diego, CA, USA). Next-generation sequencing was performed on all non-invasive samples, including those with suboptimal WGA results (i.e., weak electrophoretic band, DNA concentration <20 ng/µL) and on all but two minimally invasive samples; for these two samples, which were not sequenced, no electrophoretic band was observed, and their concentrations corresponded to the concentration level of the negative control. BlueFuse Multi software (v4.5) (Illumina, San Diego, CA, USA) was used to analyze sequencing results. The samples with mosaicism level >20% (copy numbers <1.8 and >2.2 for autosomes) were considered as aneuploid. To further characterize the aneuploid samples, we calculated the number and percentage of all aneuploids, and we separately calculated aneuploids with incorrect copy numbers involving more than 3 chromosomes (complex aneuploid), high-level mosaic samples (mosaicism level >50–<80%, copy numbers from >1.2 to <1.5 and >2.5 to <2.8), and low-level mosaic samples (mosaicism level >20–50%, copy numbers from 1.5 to <1.8 and from >2.2 to 2.5).

The quality of sequencing for each sample was inspected based on analysis of (i) the overall sample noise (DLR, derivative log ratio) and (ii) the number of reads after filtering. Overall noise (DLR) measures the spread of the difference between all bins of a chromosome (in a copy number values). In the PGT-A protocol, the optimal value of this parameter is around 0.2 (acceptance value <0.4), and values above 0.4 indicate low-quality DNA or problems during the amplification steps. The number of reads after filtering is the number of aligned and filtered reads that can be used for copy number calling. In the PGT-A procedures, the optimal number of reads after filtering is 500,000, while 250,000 is the minimum acceptable value of this parameter. Samples with low numbers of reads after filtering may present profiles that are difficult to interpret.

### 2.4. Statistical Analysis

Categorical variables were analyzed with Pearson’s χ2 or two-sided Fisher exact test, as appropriate. Analyses of the distribution of continuous data were performed using the Shapiro–Wilk test. The Mann–Whitney U test and Kruskal–Wallis test, with a post hoc test for pairwise multiple comparison, were applied to quantitative variables, as appropriate. *p* values < 0.05 were considered to be significant. Kappa statistics were used to evaluate the agreement between results obtained for non-invasive/minimally invasive samples with the results of TE-based PGT-A (current gold standard). Statistical analyses were performed with STATISTICA v.13 (StatSoft, Krakow, Poland).

## 3. Results

### 3.1. Whole-Genome Amplification and Next-Generation Sequencing Quality Data for SCM, BF+SCM, and TE

After the optimization study, all collected sample types, i.e., invasive samples (TE) and corresponding non-invasive/minimally invasive specimens (SCM, BF+SCM), were subjected to whole-genome amplification, and the results of amplification were compared between the non-invasive and minimally invasive sample types, as well as between non-invasive/minimally invasive and invasive TE samples (Table 2).

It was observed that the rate of amplification failure was lower for SCM samples than for BF+SCM samples derived from the same embryos cultured until day 5; however, the difference did not reach the level of statistical significance. A similar trend was seen for samples obtained from embryos cultured until day 6, and the difference in the amplification failure rate between SCM and BF+SCM samples was statistically significant only when all samples were analyzed together. Of note, amplification failure rates for both SCM and BF+SCM samples were not significantly different from the amplification failure rate observed among TE samples, regardless of the embryo culture duration (Table 2).

Among all successfully amplified samples, median DNA concentrations evaluated after whole-genome amplification were significantly higher for SCM samples than for BF+SCM samples (Table 2). This observation was also true for samples collected on the 6th day of culture, and a similar trend was found for samples collected on day 5, although it did not reach the level of statistical significance. Interestingly, while DNA concentrations of BF+SCM samples were significantly lower than DNA concentrations of invasive TE samples, irrespective of culture duration, SCM specimens obtained from 6-day cultures demonstrated significantly higher DNA concentrations than those observed for TE samples.

After WGA, all amplified invasive and non-invasive/minimally invasive samples were subjected to NGS-based aneuploidy testing, and the NGS quality parameters were compared between different sample types. The median overall noise (DLR) of NGS was significantly lower among SCM samples in comparison to BF+SCM samples, for both 5-day and 6-day cultures (Table 2). Nevertheless, overall noise values of NGS were the lowest among TE samples. The median number of NGS reads after filtering was the highest among invasive TE samples, and again, it was significantly different from the median number observed for SCM and BF+SCM samples, independent of culture duration. However, the comparison of the NGS reads after filtering revealed no significant differences between SCM and BF+SCM samples.

Analysis of NGS aneuploidy testing results showed that among both the SCM and BF+SCM samples from embryos cultured until day 5, there were significantly more samples with aneuploid results compared to TE samples (Table 2). This trend was also seen for complex aneuploid results, i.e., copy number gains or losses affecting more than three chromosomes, among samples from the 5-day cultures. However, among samples derived from embryos cultured until day 6, SCM and TE samples had the same rate of aneuploid results and similar rates of complex aneuploid results; meanwhile, significantly higher rates of aneuploidy, as well as complex aneuploidy, were observed for BF+SCM samples compared to both SCM and TE samples, probably reflecting the lower DNA concentration and poorer quality of the BF+SCM samples. Insight into the distribution of sex chromosomes showed that there were no significant differences in the profiles of sex chromosomes between the SCM and TE samples obtained from 5- and 6-day cultures. However, again, for BF+SCM samples, the distribution of sex chromosomes was significantly different from the distributions in the SCM and TE samples, mainly due to the higher proportion of incorrect copy numbers of sex chromosomes in BF+SCM samples; these differences were seen among samples retrieved from embryos cultured for both 5 and 6 days. The rates of high-level and low-level mosaic aneuploid results were comparable between different sample types (TE, SCM, BF+SCM), regardless of culture duration (Table 2).

### 3.2. Concordance of SCM and BF+SCM Results with Results of Corresponding TE Samples

The SCM and BF+SCM samples were then analyzed for the concordance of aneuploidy testing results with those of conventional PGT-A based on the corresponding TE sample (Table 3).

First, the overall ploidy concordance of ni/miPGT-A results with the TE-based PGT-A was estimated. Analyses of concordance between SCM and TE samples were possible for 141 cases that had both results available; 118 (83.7%) of them had concordant results (i.e., results were either euploid or aneuploid according to both sample types). The concordance between BF+SCM and TE samples was evaluated for 131 eligible cases, and there were 76 (58%) BF+SCM samples with concordant ploidy results. The concordance with TE was significantly higher for SCM samples than for BF+SCM samples (*p* < 0.001; OR = 3.71; 95% CI: 2.11–6.54). The rates of concordance for sex chromosomes and full chromosome concordance (all chromosomes in agreement between euploid or aneuploid ni/miPGT-A and PGT-A samples) were significantly higher among SCM than BF+SCM samples. Consequently, the partial chromosome concordance between non-invasive/minimally invasive samples and corresponding TE samples (at least one aberrant chromosome in agreement between aneuploid ni/miPGT-A and PGT-A) was less common for SCM than BF+SCM (Table 3).

The rate of embryos concordantly recognized as euploid based on SCM and TE (truly negative, TN) was significantly higher than the rate of embryos with concordant euploid result in BF+SCM and TE samples (Table 3). The frequency of falsely positive results (i.e., aneuploid in ni/miPGT-A and euploid in TE PGT-A, FP) was lower for SCM samples than BF+SCM. There were no significant differences between SCM and BF+SCM in terms of the rates of falsely negative (euploid in ni/miPGT-A and aneuploid in TE PGT-A, FN) and truly positive results (aneuploid both in ni/miPGT-A and TE PGT-A, TP). Among discordant ni/miPGT-A results, the false positives were more frequent than false negatives for both SCM and BF+SCM samples. The agreement between SCM and TE was characterized as substantial (Kappa = 0.673), while the agreement between BF+SCM and TE was slight (Kappa = 0.162) (Table 3).

### 3.3. Embryo Culture Duration (5 vs. 6 Days) and the Results of Non-Invasive PGT-A

Next, to investigate the potential impact of embryo culture duration on the outcome of ni/miPGT-A, we performed comparisons of quality data and aneuploidy testing results between samples obtained from embryos cultured until day 5 and day 6. Since, according to earlier analyses, SCM samples were recognized as more suitable than BF+SCM to be used in ni/miPGT-A, we show the results of comparisons between day 5 and 6 for SCM samples exclusively. DNA concentrations among SCM samples collected from 6-day cultures were significantly higher than DNA concentrations among samples from 5-day cultures (day 5: 23.9 vs. day 6: 33.6; *p* <0.001) (Table 4).

There were no significant differences between SCM samples from 5- and 6-day cultures, in terms of overall noise and number of reads in NGS after filtering. However, when the rates of SCM samples with noise values ≥0.4 were examined in both groups, it was found that these samples were significantly more frequent in the group of embryos cultured until day 5 (day 5: 14/69 (20.3%) vs. day 6: 4/73 (5.5%); *p* < 0.011; OR = 4.39; 95% CI: 1.37–14.1). Analyses of concordance of aneuploidy testing between SCM and TE samples from the groups with different embryo culture durations indicated that the rates of concordant results were lower in the group of embryos cultured until day 5. The observed lower rates of concordance among the 5-day culture group in comparison to the 6-day culture group were visible for the overall ploidy concordance and sex chromosome concordance, although the difference did not reach the level of statistical significance in either case. Nevertheless, the rates of full chromosome concordance were significantly lower in the group with 5-day blastocysts compared to 6-day embryos. Similarly, the rates of concordantly euploid embryos between SCM and TE methods (TN) were significantly lower among 5-day cultures than among embryos cultured until day 6. When discordant cases were considered, the rate of false positive results was significantly higher among SCM samples derived from 5-day cultures than among samples from 6-day cultures. All detected false negatives belonged to the group including embryos cultured for 6 days (5/72 (6.9%)). In these five cases, embryos that were evaluated as euploid according to SCM were considered aneuploid after examination of their corresponding TE samples; however, all copy number variations detected in TE cells were not full gains or losses, but mosaic changes (with an intermediate copy number between 2 and 3, or 1 and 2). Among them, there were four TE samples with low-level mosaicism (from 30% to 50%), and only one case with a high level of mosaic gain (60%) of chromosome 21. In the four samples with low-level mosaicism, the mosaic results involved only a part of a single chromosome (segmental mosaic gain of short arm of chromosome 4 observed in one sample), a whole single chromosome (a single mosaic gain of chromosome 9 detected in one sample), or two or three chromosomes. All five of these false negative cases were recorded in four couples with normal karyotypes, and in all five cases, the sex chromosomes were concordant among sampling methods, with XX chromosomes detected in four cases and XY chromosomes in one case. Additionally, one out of five embryos with a mosaic genotype detected in TE and euploid according to SCM was successfully transferred. In this particular case, an embryo with XX chromosomes was transferred, and ultrasound revealed female genitalia in the fetus.

The results of the comparisons between SCM samples collected on day 5 and 6 presented in Table 4 were not affected by the proportion of aneuploid embryos (according to the gold-standard TE-based PGT-A), as this proportion was similar in both groups (day 5: 36/70 (51.4%) vs. day 6: 35/73 (47.9%); *p* < 0.867). Since a more favorable outcome of aneuploidy testing in the group of embryos cultured until day 6 could presumably be linked to a higher rate of class AA embryos in this group (day 5: 35/70 (50%) vs. day 6: 54/73 (74%); *p* = 0.004; Table 1), we have performed the same comparisons of aneuploidy testing data between SCM samples collected from 5-day and 6-day cultures with class AA embryos separated from both groups. The results of both comparisons were similar, with the same parameters reaching the level of significance (Table 4 and Table S2). Among the AA class embryos, again DNA concentrations and rates of concordance of aneuploidy test results with TE were higher in the group of SCM samples derived from embryos cultured for 6 days compared to samples from 5-day cultures. Moreover, the agreement between SCM and TE for AA class embryos cultured until day 5 was still moderate (Kappa = 0.548), while the agreement between SCM and TE samples from 6-day cultures was almost perfect (Kappa = 0.840). Thus, the overall beneficial effect of extended embryo cultures on niPGT-A results was not driven by a higher prevalence of class AA embryos within the group of 6-day cultures, as this effect was also observed among SCM samples derived solely from class AA embryos.

### 3.4. Comparison of Concordant vs. Discordant Cases

Finally, factors that could be associated with concordance between the SCM- and TE-based aneuploidy tests results were selected and compared to analyze concordant and discordant results (Table 5).

Analyses performed for the entire ni/miPGT-A group showed that concordant cases were characterized by significantly higher median DNA concentrations in SCM samples after WGA, as well as lower overall noise values in NGS, compared to discordant cases. Among concordant cases, the rate of euploid samples by SCM was higher than that among discordant cases; however, the observed difference was not statistically significant. SCM samples with low-level mosaic aneuploid results, and those with higher numbers (>3) of aneuploid chromosomes were significantly less frequent among concordant than among discordant cases. Finally, SCM samples obtained from couples with aberrant karyotypes were more likely to be concordant with TE, with over 40% of these concordant cases attributed to truly euploid samples. When the same comparisons were restricted to the group of embryos cultured for 6 days, there were no significant differences observed between concordant and discordant cases, with the exception of SCM samples from patients with an abnormal karyotype. Moreover, there was no link between maternal and paternal age, poor sperm quality, class of embryo, the number of NGS reads after filtering, and the concordance of the PGT-A results, neither in the entire ni/miPGT-A group nor in the group of embryos cultured for 6 days (Table 5).

## 4. Discussion

Over the past decade, the concept of preimplantation genetic testing without the need for an embryo biopsy has been proposed and examined in several studies [12,13,14,16,17,18,19,20,21,22,23,24,25,26,27,28,29,30,31,32,33,34]. Despite inconsistent conclusions drawn from prior studies on the use of non-invasive/minimally invasive samples in PGT-A, pointing to the need for additional research before these methods can be adopted for clinical use, commercial niPGT-A assays have been developed and are now available for IVF patients. On the other hand, Hanson et al. presented data from 166 embryos that were tested using such a commercial assay, showing that the results were unsatisfactory in terms of both amplification failure rate (37.3%) and ploidy concordance with TE (63.5%) [26,36]. It was also reported that the concordance of the results obtained using commercial niPGT-A assay with traditional TE-based PGT-A results is clearly affected by the culture conditions and duration [38].

Thus, we aimed to develop non-invasive/minimally invasive PGT-A procedures that could be used in a clinical setting and that would be as reliable as the current gold-standard PGT-A technique performed with trophectoderm biopsy. Particularly, we intended to determine the optimal ni/miPGT-A approach in terms of sample type and sampling day. In our study, amplification failure rates for non-invasive and minimally invasive sample types (SCM and BF+SCM) were comparable to the amplification failure rates for trophectoderm, indicating that the former can be used in preimplantation genetic testing with a similar risk of not obtaining results as the routinely used TE. The rate of uninformative non-invasive SCM samples observed in our study with two-step fresh embryo cultures was 0.7% (1/143, Table 2), which is one of the lowest among failure rates previously reported, which range from 0% [12] to 37.3% [12,26] depending on the culture conditions, duration of culture, and WGA method applied.

We found that compared to SCM, the amplification failure rate determined for minimally invasive BF+SCM was higher (7.7%, 11/143, Table 2), so it might be concluded that there is no additional benefit from combining SCM with blastocyst fluid obtained by blastocyst collapse, which was expected to contain additional cfDNA, and thus should allow for more efficient amplification. The higher amplification failure rate of BF+SCM in our study may be related to the technique used to collect this type of sample, in which the collapse of the blastocyst and the release of its contents was not performed directly in the SCM but in fresh medium, and both fluids (medium with released blastocoel fluid and SCM) were combined afterwards, resulting in unfavorable dilution of cfDNA. Additionally, the higher rate of amplification failure among BF+SCM samples was in line with the overall worse performance of this sample type. Since the BF+SCM samples in our study, compared to SCM and TE, had the lowest concentrations of DNA after WGA, had the noisiest NGS profiles, and were more likely to be aneuploid (including complex aneuploidy), it is possible that the results observed for BF+SCM may reflect an insufficient quantity and poor quality of DNA in these samples, underlying the high false-positive rate and poor concordance with TE. Although the rate of uninformative minimally invasive BF+SCM samples observed in our study was within the range reported in previous studies [9,16,27,28,29,30,31,33], the rate of overall ploidy concordance with TE results for BF+SCM was apparently lower (58%, 76/131, Table 3) than the rates determined before, ranging from 76.3% (29/38) [28] to 100% (26/26) in a study including re-cultured vitrified blastocysts donated for research [16]. However, according to our data, BF+SCM retrieved from fresh embryo culture is not a suitable sample for use in preimplantation genetic testing for aneuploidy.

An additional consideration regarding the observed variability in cfDNA amplification success and concentration is the potential impact of embryo quality. High-rated embryos may possess stronger tight junctions, which are essential for maintaining cellular cohesion and preventing the leakage of intracellular components, including cfDNA, into the surrounding culture medium [39]. This could lead to lower cfDNA concentrations and potentially reduce the efficiency of cfDNA amplification and subsequent genetic analyses. In contrast, lower-rated embryos, often associated with increased levels of apoptosis, may release greater amounts of cfDNA due to higher cellular turnover and structural compromise [40,41]. These biological differences could explain some of the variability in cfDNA results observed across different embryos and merit further investigation to improve the reliability and interpretability of non-invasive genetic testing methods.

On the other hand, the lower amplification failure rates, higher DNA concentrations after WGA, and lower NGS overall noise values among SCM samples in comparison to the corresponding BF+SCM samples (Table 2) strongly suggested that SCM is the more adequate sample type for use in ni/miPGT-A. The usefulness of SCM for niPGT-A was further confirmed by comparisons of DNA concentrations with the corresponding TE and the inspection of NGS quality parameters for SCM samples relative to TE samples. The DNA concentrations after WGA were comparable between all SCM and TE samples and were even significantly higher among SCM than in TE samples collected from embryos cultured until day 6. Although overall noise values in NGS were significantly higher for SCM in comparison to TE, still over 85% of all SCM samples (124 of 142 successfully amplified) and 95% of SCM samples from embryos cultured until day 6 (69/73) had noise values less than 0.4, which is considered an acceptable level in PGT-A procedures. Similarly, while the number of reads after filtering were the highest for TE samples, still over the half of SCM samples had a number higher than the PGT-A acceptance level of 250,000 (59.2% (84/142) for all SCM samples, and 57.5% (42/73) for SCM collected from 6-day cultures). Overall, this suggests that while SCM samples may have lower NGS quality in comparison to TE, the majority of SCM samples will present similar NGS quality to routinely used TE cells.

This study showed that 83.7% of SCM samples (118/141, Table 3) had concordant overall ploidy results with the reference TE samples. Higher ploidy concordance rates for SCM samples were reported by Huang et al. (89.1%, 41/46) [13] and Shitara et al. (88.9%, 16/18) [22] in studies conducted with vitrified embryos donated for research, while most previous studies had SCM-TE concordance rates of less than 80% [17,18,19,20,21,23,24,26,38]. However, it has previously been shown that concordance rates can be improved when spent culture media are obtained from embryos cultured for 6 days in comparison to those cultured for 5 days [20,38]. A similar enhancement of concordance rates by prolonging the embryo cultures from 5 to 6 days was also observed in our study, with an increase in concordance from 79.7% for SCM samples collected on day 5 to 87.5% among SCM samples collected on day 6 (Table 4). Other reported benefits of extending the duration of embryo culture to 6 days included the ability to obtain higher DNA concentrations and higher rates of informative NGS results among SCM samples collected on day 6 compared to samples collected on day 5 [24]. In our study, we were able to confirm that post-amplification DNA concentrations were significantly higher for SCM samples collected on day 6 than those collected on day 5, but due to the very low amplification failure rate, we could not observe significant differences in the informativity rates between SCM samples collected on day 5 and day 6. Of note, the additional day of embryo culture, favorable in terms of niPGT-A results, did not seem to negatively affect the reproductive capacity of the embryos, as the fractions of blastocysts that were eligible for transfer (i.e., euploid according to TE testing) were comparable between the 5- and 6-day cultures (day 5: 34/70, 48.6% vs. day 6: 37/73, 50.7%; *p* = 0.867). In summary, our data indicate that SCM samples obtained from embryo cultures conducted for 6 days can provide the most appropriate niPGT-A results in terms of informativity rates and reliability of aneuploidy testing. However, there are currently no data on the impact of extended culture durations on the clinical outcome for day 6 embryo transfers performed with the non-invasive PGT-A.

Despite the fact that SCM samples obtained from embryos cultured for 6 days showed the highest rate of concordant results with those of the corresponding TE samples, there was still a non-negligible fraction of discordant results deserving special attention due to the potential serious consequences for the outcomes of IVF procedures. Among all cases with discordant results between SCM-based niPGT-A and TE PGT-A results, false negatives were less frequent than false positives (3.5% vs. 12.8%); nevertheless, all false negative results were observed in the group of embryos cultured until day 6. In all cases of false negative SCM results, embryos were deemed to be mosaic according to TE, with four out of five showing low-level mosaicism (>20–50%). In fact, mosaicism detected by next-generation sequencing techniques in TE samples is considered a poor predictor of ploidy status of the rest of the embryo, and the actual ploidy status of mosaic samples cannot be reliably determined in a clinical setting. Thus, such “putatively mosaic” samples may be euploid, aneuploid, or true mosaic [42]. For example, it was reported that 85.4% (35/41) of “putative mosaic” blastocysts and all blastocysts classified as low-level (20–50%) mosaics according to TE testing were identified as euploid after being retested using the remaining blastocyst embryo [28]. It was also shown that mosaicism levels under 50% in a TE biopsy usually reflect a status of highly confined aneuploidy, with the rest of the embryo being unaffected. Subsequently, investigators have reported that developmental potential and clinical outcomes of such mosaic embryos may be similar to those of uniformly euploid ones [43]. Furthermore, despite the fact that more than 2700 embryos with mosaic PGT-A results have been transferred to date, leading to hundreds of live births, only a few cases of mosaicism or aneuploidy persistence after delivery have been reported [11,44,45]. Also in our study, one out of five embryos with mosaic genotypes detected in TE and detected as euploid according to SCM was transferred, and a positive outcome was observed, further confirmed by normal third-trimester ultrasound results. In this light, false negative ni/miPGT-A results may be less problematic than false positives, which should be of greater concern.

Although the rate of false positives was reduced in the group of embryos cultured for 6 days, compared to the group with 5 days of incubation, still 5.6% (4/72) of SCM samples were falsely found to be aneuploid, restricting the number of embryos suitable for transfer when using niPGT-A to select euploid embryos. In many studies, the rate of false positivity was similar to, or even higher than, the rate established in our study [17,19,20,21,24,26]. In addition, our attempts to detect possible factors underlying the discordant results within the group of SCM samples from cultures conducted for 6 days were unsuccessful (Table 5). Thus, in order to avoid the loss of euploid embryos, falsely recognized as aneuploid based on SCM testing, we propose to verify positive (aneuploid) niPGT-A results by introducing an additional step of testing trophectoderm cells, after obtaining the patients’ informed consent.

Another issue with serious practical implications is the discordances in sex chromosomes determined by analysis of DNA in SCM (niPGT-A) and TE (PGT-A). Such discrepancies were reported in a number of previous studies [18,19,20,21,22,23,24,26,38] and were attributed to contamination of non-invasive samples with DNA of maternal origin, since usually embryos detected to be male based on TE were misdiagnosed as female by analysis of the corresponding non-invasive sample. In our study, there were 21 SCM samples (14 from 5-day embryo cultures, 7 from 6-day embryo cultures) with discordant sex chromosomes determined by TE, of which all but 2 were aneuploid according to SCM. These two euploid SCM samples collected from embryos cultured for 5 days were found to be euploid XY by TE and euploid XX by SCM, suggesting possible contamination with maternal DNA. However, in the group of embryos cultured for 6 days, all seven cases with discordant sex chromosomes were aneuploid according to SCM, and six of them had incorrect numbers of sex chromosomes. Given that embryos detected as aneuploid by the SCM testing could be retested with the use of TE biopsy, the risk of sex chromosome misdiagnosis is reduced, albeit not entirely eliminated for euploid embryos. Thus, niPGT-A should not be used to select female embryos in case the mother is a carrier of X-linked recessive disease.

The proper interpretation and decision making regarding the transfer of embryos with mosaic niPGT-A results are at least as difficult as in the case of mosaic TE-based PGT-A results, given that there are no published data on transfers of embryos deemed mosaic based on non-invasive samples. Hence, it should be noted that the percentage of mosaic niPGT-A results obtained through SCM testing was not higher than that obtained using TE. In the group of SCM samples from cultures conducted for 6 days, there were only four cases with mosaic niPGT-A results.

The small overall sample size in this study is a main limitation. Further steps that should be performed to clarify the utility of non-invasive PGT-A include its extended analytical validation, clinical validation in non-selection trials to determine the positive and negative predictive values of niPGT-A results, and, finally, randomized controlled trials comparing outcomes of embryo transfers after niPGT-A with those of transfers without genetic testing or guided by conventional PGT-A.

## 5. Conclusions

The results of our study add more information to the current fragmentary knowledge on the capacity of ni/miPGT-A techniques and show that, under certain conditions, SCM may serve as a safe, minimally invasive approach to perform preimplantation genetic testing for aneuploidy. The next step warranted in research on non-invasive PGT-A is an extensive assessment of the effectiveness of using niPGT-A to select embryos suitable for transfer, including evaluation of live birth and miscarriage rates.

## 6. Patents

Gyncentrum Sp. z o. o., Katowice, Poland, applied to the patent office for a method of non-invasive preimplantation genetic testing of embryos. Application number P.442092 was assigned on 25 August 2022 and has the pending status.

## Figures and Tables

**Table 1:** Comparison of patients and embryos from the groups with different embryo culture duration.

Characteristics	ni/miPGT-A Group—Day 5 N = 15 (15 IMSI Cycles)	ni/miPGT-A Group—Day 6 N = 16 (16 IMSI Cycles)	*p*
Age of female partner ^a^	36 (33–38)	36 (32–37)	0.796 ^b^
Age of male partner ^a^	38 (35–41)	38 (34–39.5)	0.780 ^b^
Women ≥ 35 years old	10 (66.7%)	10 (62.5%)	1.000 ^c^
Couples with severe male factor	2 (13.3%)	3 (18.8%)	1.000 ^c^
Couples with previous unsuccessful IVF history	9 (60%)	6 (37.5%)	0.289 ^c^
Couples with previous unsuccessful IUI history	9 (60%)	6 (37.5%)	0.289 ^c^
Patients with aberrant karyotype	2 (13.3%) 46,XX,t(5;18) 45,XX,rob(13;14)(q10;q10)	3 (18.8%) 45,XX,rob(14;21)(q10;q10) 45,X [15]/46.XX [53] 47,XYY	1.000 ^c^
Number of MII oocytes fertilized by IMSI	132 (from 4 to 14/IMSI cycle)	127 (from 2 to 15/IMSI cycle)	
Number of viable embryos	77 (58.3%) (from 2 to 10/IMSI cycle)	96 (75.6%) (from 1 to 15/IMSI cycle)	0.004 ^c^
Number of embryos with no development potential	55 (41.7%) (from 0 to 8/IMSI cycle)	31 (24.4%) (from 0 to 6/IMSI cycle)	
Number of viable embryos subjected to PGT-A analysis	70 (from 2 to 8/IMSI cycle)	73 (from 1 to 15/IMSI cycle)	
**Embryos’ class according to Gardner system [N (%)]**			
AA	35 (50%)	54 (74%)	0.004 ^c^ AA vs. AB&BB
AB	22 (31.4%)	17 (23.3%)	
BB	13 (18.6%)	2 (2.7)	

^a^—median (lower and upper quartile); ^b^—*p* value of Mann–Whitney U test; ^c^—*p* value of two-tailed Fisher’s exact test. ni/miPGT-A—non-invasive/minimally invasive preimplantation genetic testing for aneuploidy; IMSI—intracytoplasmic morphologically selected sperm injection; IVF—in vitro fertilization; IUI—intrauterine insemination.

**Table 2:** Comparison of whole-genome amplification (WGA) outcome and aneuploidy testing quality parameters and results for trophectoderm (TE)-biopsied cells and two non-invasive/minimally invasive sample types (spent culture medium (SCM) and a mixture of blastocoel fluid and spent culture medium (BF+SCM)) obtained from embryos cultured until day 5 and day 6.

Embryo Samples	TE	SCM	BF+SCM		*p*	
				SCM vs. BF+SCM	SCM vs. TE	BF+SCM vs. TE
**ni/miPGT-A group—day 5 (N = 70)**						
Samples with amplification failure	4 (5.7%) ^a^	1 (1.4%)	6 (8.6%)	0.116 ^b^	0.366 ^b^	0.745 ^b^
DNA concentration [ng/µL] ^c^	28.2 (24.4–34.2)	23.9 (21.0–28.4)	22.6 (19.5–25.8)	0.172 ^d^	<0.001 ^d^	<0.001 ^d^
NGS overall noise (DLR) ^c^	0.19 (0.18–0.24)	0.29 (0.21–0.38)	0.41 (0.30–0.59)	<0.001 ^d^	<0.001 ^d^	<0.001 ^d^
Number of NGS reads after filtering ^c^	536,402 (423,572–610,572)	315,857 (150,368–434,621)	309,454 (144,916–482,065)	1.000 ^d^	<0.001 ^d^	<0.001 ^d^
**Result of genetic testing for aneuploidy**						
Aneuploid, all	36 (51.4%)	50 (71.4%)	55 (78.6%)	0.435 ^b^	0.024 ^b^	0.001 ^b^
Complex aneuploid (>3 chromosomes)	7 (10%)	18 (25.7%)	29 (41.4%)	0.073 ^b^	0.026 ^b^	<0.001 ^b^
High-level mosaic samples (>50–<80%)	1 (1.4%)	5 (7.1%)	6 (8.6%)	1.000 ^b^	0.209 ^b^	0.116 ^b^
Low-level mosaic samples (>20–50%)	8 (11.4%)	8 (11.4%)	5 (7.1%)	0.562 ^b^	1.000 ^b^	0.562 ^b^
No result	0	1 (1.4%)	6 (8.6%)	0.116 ^b^	1.000 ^b^	0.028 ^b^
**Sex chromosomes**						
XX	31 (44.3%)	31 (44.3%)	21 (30%)	0.046 ^e^	0.690 ^e^	0.006 ^e^
XY	31 (44.3%)	27 (38.6%)	21 (30%)			
Incorrect	8 (11.4%)	11 (15.7%)	22 (31.4%)			
**ni/miPGT-A group—day 6 (N = 73)**						
Samples with amplification failure	2 (2.7%) ^a^	0	5 (6.8%)	0.058 ^b^	0.497 ^b^	0.442 ^b^
DNA concentration [ng/µL] ^c^	29.1 (27.6–31.4)	33.6 (30.8–36.2)	27.8 (24.9–30.3)	<0.001 ^d^	<0.001 ^d^	0.025 ^d^
NGS overall noise (DLR) ^c^	0.20 (0.18–0.22)	0.25 (0.23–0.30)	0.50 (0.36–1.11)	<0.001 ^d^	<0.001 ^d^	<0.001 ^d^
Number of NGS reads after filtering ^c^	410,850 (351,654–490,207)	262,840 (215,526–322,930)	264,766 (150,247–377,737)	1.000 ^d^	<0.001 ^d^	<0.001 ^d^
**Result of genetic testing for aneuploidy**						
Aneuploid, all	35 (47.9%)	35 (47.9%)	61 (83.6%)	<0.001 ^b^	1.000 ^b^	<0.001 ^b^
Complex aneuploid (>3 chromosomes)	5 (6.8%)	6 (8.2%)	33 (45.2%)	<0.001 ^b^	1.000 ^b^	<0.001 ^b^
High-level mosaic samples (>50–<80%)	3 (4.1%)	1 (1.4%)	3 (4.1%)	0. 620 ^b^	0. 620 ^b^	1.000 ^b^
Low-level mosaic samples (>20–50%)	8 (11%)	3 (4.1%)	2 (2.7%)	1.000 ^b^	0.208 ^b^	0.097 ^b^
No result	1 (1.4%)	0	5 (6.8%)	0.058 ^b^	1.000 ^b^	0.209 ^b^
**Sex chromosomes**						
XX	41 (56.2%)	36 (49.3%)	26 (35.6%)	<0.001 ^e^	0.211 ^e^	<0.001 ^e^
XY	29 (39.7%)	30 (41.1%)	16 (21.9%)			
Incorrect	2 (2.7%)	7 (9.6%)	26 (35.6%)			
**ni/miPGT-A group, all samples (N = 143)**						
Samples with amplification failure	6 (4.2%) ^a^	1 (0.7%)	11 (7.7%)	0.005 ^b^	0.120 ^b^	0.317 ^b^
DNA concentration [ng/µL] ^c^	28.8 (26.5–32.0)	29.8 (23.7–33.7)	25.5 (21.4–28.5)	<0.001 ^d^	1.000 ^d^	<0.001 ^d^
NGS overall noise (DLR) ^c^	0.19 (0.18–0.22)	0.26 (0.22–0.33)	0.46 (0.32–0.80)	<0.001 ^d^	<0.001 ^d^	<0.001 ^d^
Number of NGS reads after filtering ^c^	473,087 (373,698–559,876)	286,581 (184,304–370,756)	286,472 (147,524–405,540)	1.000 ^d^	<0.001 ^d^	<0.001 ^d^
**Result of genetic testing for aneuploidy**						
Aneuploid, all	71 (49.7%)	85 (59.4%)	116 (81.1%)	<0.001 ^b^	0.123 ^b^	<0.001 ^b^
Complex aneuploid (>3 chromosomes)	12 (8.4%)	24 (16.8%)	62 (43.4%)	<0.001 ^b^	0.049 ^b^	<0.001 ^b^
High-level mosaic samples (>50–<80%)	4 (2.8%)	6 (4.2%)	9 (6.3%)	0.597 ^b^	0.750 ^b^	0.256 ^b^
Low-level mosaic samples (>20–50%)	16 (11.2%)	11 (7.7%)	7 (4.9%)	0.466 ^b^	0.419 ^b^	0.080 ^b^
No result	1 (0.7%)	1 (0.7%)	11 (7.7%)	0.005 ^b^	1.000 ^b^	0.005 ^b^
**Sex chromosomes**						
XX	72 (50.3%)	67 (46.9%)	47 (32.9%)	<0.001 ^e^	0.280 ^e^	<0.001 ^e^
XY	60 (42%)	57 (39.9%)	37 (25.9%)			
Incorrect	10 (7%)	18 (12.6%)	48 (33.6%)			

^a^—embryos with amplification failure observed for TE samples were subjected to TE rebiopsy and second WGA (second WGA was not successful for only one embryo cultured until day 6); ^b^—*p* value of two-tailed Fisher’s exact test; ^c^—median (lower and upper quartile) for all samples without amplification failure; ^d^—*p* value of the pairwise multiple comparison for the Kruskal–Wallis test; ^e^—*p* value of Pearson’s Chi-square test. ni/miPGT-A—non-invasive/minimally invasive preimplantation genetic testing for aneuploidy; TE—trophectoderm; SCM—spent culture medium; BF—blastocoel fluid; NGS—next-generation sequencing; DLR—derivative log ratio.

**Table 3:** Concordance of non-invasive/minimally invasive PGT-A results with TE-based PGT-A for 141 SCM and 131 BF+SCM samples with both informative results available.

Results	SCM (N = 141)	BF+SCM (N = 131)	*p* ^ a^	OR (95% CI)
**Ploidy concordance of ni/miPGT-A with PGT-A: ^b^**				
Concordant	118 (83.7%)	76 (58%)	<0.001	3.71 (2.11–6.54)
Discordant	23 (16.3%)	55 (42%)		
**Chromosome concordance of ni/miPGT-A with PGT-A:**				
Sex chromosomes	120 (85.1%)	73 (55.7%)	<0.001	4.54 (2.55–8.09)
Full (all chromosomes concordant) ^c^	74 (52.5%)	18 (13.7%)	<0.001	6.93 (3.82–12.60)
Partial (at least one aberrant chromosome in agreement)	36 (25.5%)	50 (38.2%)	0.027	0.56 (0.33–0.93)
None (no common aberrant chromosome between aneuploid samples)	8 (5.7%)	8 (6.1%)	1.000	-
**Concordance/discordance characteristics:**				
Euploid in ni/miPGT-A–euploid in TE PGT-A (TN)	52 (36.9%)	13 (9.9%)	<0.001	5.30 (2.72–10.33)
Aneuploid in ni/miPGT-A–aneuploid in TE PGT-A (TP)	66 (46.8%)	63 (48.1%)	0.903	-
Euploid in ni/miPGT-A–aneuploid in TE PGT-A (FN)	5 (3.5%)	2 (1.5%)	0.449	-
Aneuploid in ni/miPGT-A–euploid in TE PGT-A (FP)	18 (12.8%)	53 (40.5%)	<0.001	0.22 (0.12–0.39)
Sensitivity (relative to TE PGT-A)	93.0%	96.9%	-	-
Specificity (relative to TE PGT-A)	74.3%	19.7%	-	-
Kappa statistics	0.673 substantial agreement	0.162 slight agreement	-	-

^a^—*p* value of two-tailed Fisher’s exact test; ^b^—concordant ploidy results of ni/miPGT-A (SCM or BF+SCM) with results of PGT-A based on TE samples are defined as euploid results in both sample types or aneuploid in both sample types, while discordant ploidy results include those euploid in PGT-A and aneuploid in ni/miPGT-A, as well as aneuploid in PGT-A and euploid in ni/miPGT-A; ^c^—full chromosome concordance includes specimens with euploid results in both ni/miPGT-A and PGT-A, or aneuploid for the same chromosomes. ni/miPGT-A—non-invasive/minimally invasive preimplantation genetic testing for aneuploidy; TE—trophectoderm; SCM—spent culture medium; BF—blastocoel fluid.

**Table 4:** Comparisons of genetic testing quality parameters and rates of concordance with TE between SCM samples derived from embryos cultured for 5 and 6 days.

Parameters	SCM (niPGT-A)			
	ni/miPGT-A Group—Day 5 N = 70	ni/miPGT-A Group—Day 6 N = 73	*p*	OR (95% CI)
Samples with amplification failure	1 (1.4%)	0	0.490 ^a^	-
DNA concentration [ng/µL] ^b^	23.9 (21.0–28.4)	33.6 (30.8–36.2)	<0.001 ^c^	NA
NGS overall noise (DLR) ^b^	0.29 (0.21–0.38)	0.25 (0.23–0.30)	0.219 ^c^	NA
Number of NGS reads after filtering ^b^	315,857 (150,368–434,621)	262,840 (215,526–322,930)	0.177 ^c^	NA
Samples eligible for concordance analyses (with informative results of both niPGT-A and TE PGT-A)	N = 69	N = 72		
**Ploidy concordance of niPGT-A with PGT-A ^d^**				
Concordant	55 (79.7%)	63 (87.5%)	0.257 ^a^	-
Discordant	14 (20.3%)	9 (12.5%)		
**Chromosome concordance of niPGT-A with PGT-A**				
Sex chromosomes	55 (79.7%)	65 (90.3%)	0.099 ^a^	-
Full (all chromosomes concordant) ^e^	30 (43.5%)	44 (61.1%)	0.044 ^a^	0.49 (0.25–0.96)
Partial (at least one aberrant chromosome in agreement)	19 (27.5%)	17 (23.6%)	0.700 ^a^	-
None (no common aberrant chromosome between aneuploid samples)	6 (8.7%)	2 (2.8%)	0.160 ^a^	-
**Concordance/discordance characteristics**				
Euploid in niPGT-A–euploid in TE PGT-A (TN)	19 (27.5%)	33 (45.8%)	0.036 ^a^	0.45 (0.22–0.91)
Aneuploid in niPGT-A–aneuploid in TE PGT-A (TP)	36 (52.2%)	30 (41.7%)	0.240 ^a^	-
Euploid in niPGT-A–aneuploid in TE PGT-A (FN)	0	5 (6.9%)	0.058 ^a^	-
Aneuploid in niPGT-A–euploid in TE PGT-A (FP)	14 (20.3%)	4 (5.6%)	0.011 ^a^	4.33 (1.35–13.90)
Sensitivity (relative to TE PGT-A)	100%	85.7%		
Specificity (relative to TE PGT-A)	57.6%	89.2%		
Kappa statistics	0.586 moderate agreement	0.750 substantial agreement		

^a^—*p* value of two-tailed Fisher’s exact test; ^b^—median (lower and upper quartile); ^c^—*p* value of Mann–Whitney U test; ^d^—concordant ploidy results of niPGT-A (SCM) with results of PGT-A based on TE samples are defined as euploid results in both sample types or aneuploid in both sample types, while discordant ploidy results include those euploid in PGT-A and aneuploid in niPGT-A, as well as aneuploid in PGT-A and euploid in niPGT-A; ^e^—full chromosome concordance includes specimens with euploid results in both niPGT-A and PGT-A or aneuploid for the same chromosomes. ni/miPGT-A—non-invasive/minimally invasive preimplantation genetic testing for aneuploidy; TE—trophectoderm; SCM—spent culture medium; NGS—next-generation sequencing; DLR—derivative log ratio.

**Table 5:** Characteristics of cases with concordant and discordant results between ni/miPGT-A (based on SCM) with TE PGT-A.

SCM ni/miPGT-A Group Results	SCM ni/miPGT-A Group—Day 6 Results							
Characteristics	Concordant with TE N = 118	Discordant with TE N = 23	*p*	OR (95% CI)	Concordant with TE N = 63	Discordant with TE N = 9	*p*	OR (95% CI)
Age of female partner ^a^	35 (32–37)	33 (32–36)	0.288 ^b^	NA	32 (32–37)	32 (32–36)	0.568 ^b^	NA
Age of male partner ^a^	38 (35–40)	37 (33–39)	0.308 ^b^	NA	38 (33–38)	36 (33–39)	0.780 ^b^	NA
Women ≥35 years old	65 (55.1%)	9 (39.1%)	0.178 ^c^		27 (42.9%)	3 (33.3%)	0.726 ^c^	
Severe male factor	20 (16.9%)	4 (17.4%)	1.000 ^c^		14 (22.2%)	4 (44.4%)	0.214 ^c^	
Patients’ aberrant karyotype	32 (27.1%)	1 (4.3%)	0.016 ^c^	8.2 (1.06–63.3)	24 (38.1%)	0	0.025 ^c^	
DNA concentration [ng/µL] ^a^	30.7 (24.1–33.8)	23.9 (19.9–31)	0.011 ^b^	NA	33.6 (30.8–36.2)	33.4 (23.7–35.5)	0.507 ^b^	NA
NGS overall noise (DLR) ^a^	0.25 (0.21–0.32)	0.29 (0.23–0.49)	0.007 ^b^	NA	0.25 (0.22–0.30)	0.26 (0.23–0.27)	0.500 ^b^	NA
Number of NGS reads after filtering ^a^	295,738 (197,993–370,756)	256,208 (140,176–383,565)	0.236 ^b^	NA	263,958 (219,329–324,369)	256,208 (215,029–320,731)	0.973 ^b^	NA
**Embryos’ class**								
AA	75 (63.6%)	12 (52.2%)	0.589 ^d^	-	49 (77.8%)	4 (44.4%)	0.059 ^d^	-
AB	31 (26.3%)	8 (34.8%)			13 (20.6%)	4 (44.4%)		
BB	12 (10.2%)	3 (13%)			1 (1.6%)	1 (11.1%)		
**Sex chromosomes detected in TE**								
XX	57 (48.3%)	14 (60.9%)	0.269 ^d^	-	33 (52.4%)	8 (88.9%)	0.116 ^d^	-
XY	51 (43.2%)	9 (39.1%)			28 (44.4%)	1 (11.1%)		
Incorrect	10 (8.5%)	0			2 (3.2%)	0		
**SCM niPGT-A outcome**								
Euploid	52 (44.1%)	5 (21.7%)	0.062 ^c^	-	33 (52.4%)	5 (55.5%)	1.000 ^c^	-
Aneuploid, all	66 (55.9%)	18 (78.3%)			30 (47.6%)	4 (44.4%)		
High-level mosaic samples (>50–<80%)	4 (3.4%)	2 (8.7%)	0.253 ^c^	-	1 (1.6%)	0	1.000 ^c^	-
Low-level mosaic samples (>20–50%)	5 (4.2%)	5 (21.7%)	0.011 ^c^	0.16 (0.04–0.61)	1 (1.6%)	1 (11.1%)	0.236 ^c^	-
**No. of aneuploid chromosomes/sample in SCM**								
0–3	102 (86.4%)	15 (65.2%)	0.029 ^c^	3.4 (1.24–9.31)	58 (92.1%)	8 (88.9%)	0.565 ^c^	-
>3	16 (13.6%)	8 (34.8%)			5 (7.9%)	1 (11.1%)		
Median (lower and upper quartile)	1 (0–2)	2 (1–7)	0.005 ^b^	NA	0 (0–1)	0 (0–2)	0.867 ^b^	NA

^a^—median (lower and upper quartile); ^b^—*p* value of Mann–Whitney U test; ^c^—*p* value of two-tailed Fisher’s exact test; ^d^—*p* value of Pearson’s Chi-square test. ni/miPGT-A—non-invasive/minimally invasive preimplantation genetic testing for aneuploidy; TE—trophectoderm; SCM—spent culture medium; DLR—derivative log ratio. Concordant results of niPGT-A (SCM) with results of PGT-A based on TE samples are defined as euploid or aneuploid results for both sample types; discordant ploidy results include those euploid in PGT-A and aneuploid in niPGT-A, as well as aneuploid in PGT-A and euploid in niPGT-A.

## Data Availability

The original contributions presented in this study are included in the article/Supplementary Material. Further inquiries can be directed to the corresponding author.

## Supplementary Materials

The following supporting information can be downloaded at: https://www.mdpi.com/article/10.3390/jcm14010033/s1, Table S1: Patients’ and embryos’ characteristics; Table S2: Comparisons of genetic testing quality parameters and rates of concordance with TE between SCM samples derived from class AA embryos cultured for 5 and 6 days.

## References

[1] Carvalho F., Coonen E., Goossens V., Kokkali G., Rubio C., Meijer-Hoogeveen M., Moutou C., Vermeulen N., De Rycke M., ESHRE PGT Consortium Steering Committee (2020). ESHRE PGT Consortium Good Practice Recommendations for the Organisation of PGT. Hum. Reprod. Open.

[2] Homer H.A. (2019). Preimplantation Genetic Testing for Aneuploidy (PGT-A): The Biology, the Technology and the Clinical Outcomes. Aust. N. Z. J. Obstet. Gynaecol..

[3] Sciorio R., Dattilo M. (2020). PGT-A Preimplantation Genetic Testing for Aneuploidies and Embryo Selection in Routine ART Cycles: Time to Step Back?. Clin. Genet..

[4] Leaver M., Wells D. (2020). Non-Invasive Preimplantation Genetic Testing (niPGT): The next Revolution in Reproductive Genetics?. Hum. Reprod. Update.

[5] Moustakli E., Zikopoulos A., Skentou C., Bouba I., Dafopoulos K., Georgiou I. (2024). Evolution of Minimally Invasive and Non-Invasive Preimplantation Genetic Testing: An Overview. J. Clin. Med..

[6] Scott R.T., Upham K.M., Forman E.J., Zhao T., Treff N.R. (2013). Cleavage-Stage Biopsy Significantly Impairs Human Embryonic Implantation Potential While Blastocyst Biopsy Does Not: A Randomized and Paired Clinical Trial. Fertil. Steril..

[7] Guzman L., Nuñez D., López R., Inoue N., Portella J., Vizcarra F., Noriega-Portella L., Noriega-Hoces L., Munné S. (2019). The Number of Biopsied Trophectoderm Cells May Affect Pregnancy Outcomes. J. Assist. Reprod. Genet..

[8] Cimadomo D., Capalbo A., Ubaldi F.M., Scarica C., Palagiano A., Canipari R., Rienzi L. (2016). The Impact of Biopsy on Human Embryo Developmental Potential during Preimplantation Genetic Diagnosis. Biomed. Res. Int..

[9] Zhang W.Y., von Versen-Höynck F., Kapphahn K.I., Fleischmann R.R., Zhao Q., Baker V.L. (2019). Maternal and Neonatal Outcomes Associated with Trophectoderm Biopsy. Fertil. Steril..

[10] Makhijani R., Bartels C.B., Godiwala P., Bartolucci A., DiLuigi A., Nulsen J., Grow D., Benadiva C., Engmann L. (2021). Impact of Trophectoderm Biopsy on Obstetric and Perinatal Outcomes Following Frozen-Thawed Embryo Transfer Cycles. Hum. Reprod..

[11] Muñoz E., Bronet F., Lledo B., Palacios-Verdú G., Martinez-Rocca L., Altmäe S., Pla J., representing the Special Interest Group in Reproductive Genetics of the Spanish Society of Fertility (2024). To Transfer or Not to Transfer: The Dilemma of Mosaic Embryos—A Narrative Review. Reprod. Biomed. Online.

[12] Xu J., Fang R., Chen L., Chen D., Xiao J.-P., Yang W., Wang H., Song X., Ma T., Bo S. (2016). Noninvasive Chromosome Screening of Human Embryos by Genome Sequencing of Embryo Culture Medium for in Vitro Fertilization. Proc. Natl. Acad. Sci. USA.

[13] Huang L., Bogale B., Tang Y., Lu S., Xie X.S., Racowsky C. (2019). Noninvasive Preimplantation Genetic Testing for Aneuploidy in Spent Medium May Be More Reliable than Trophectoderm Biopsy. Proc. Natl. Acad. Sci. USA.

[14] Palini S., Galluzzi L., De Stefani S., Bianchi M., Wells D., Magnani M., Bulletti C. (2013). Genomic DNA in Human Blastocoele Fluid. Reprod. Biomed. Online.

[15] Shamonki M.I., Jin H., Haimowitz Z., Liu L. (2016). Proof of Concept: Preimplantation Genetic Screening without Embryo Biopsy through Analysis of Cell-Free DNA in Spent Embryo Culture Media. Fertil. Steril..

[16] Chen J., Jia L., Li T., Guo Y., He S., Zhang Z., Su W., Zhang S., Fang C. (2021). Diagnostic Efficiency of Blastocyst Culture Medium in Noninvasive Preimplantation Genetic Testing. F S Rep..

[17] Chen L., Sun Q., Xu J., Fu H., Liu Y., Yao Y., Lu S., Yao B. (2021). A Non-Invasive Chromosome Screening Strategy for Prioritizing in Vitro Fertilization Embryos for Implantation. Front. Cell Dev. Biol..

[18] Ho J.R., Arrach N., Rhodes-Long K., Ahmady A., Ingles S., Chung K., Bendikson K.A., Paulson R.J., McGinnis L.K. (2018). Pushing the Limits of Detection: Investigation of Cell-Free DNA for Aneuploidy Screening in Embryos. Fertil. Steril..

[19] Lledo B., Morales R., Ortiz J.A., Rodriguez-Arnedo A., Ten J., Castillo J.C., Bernabeu A., Llacer J., Bernabeu R. (2021). Consistent Results of Non-Invasive PGT-A of Human Embryos Using Two Different Techniques for Chromosomal Analysis. Reprod. Biomed. Online.

[20] Rubio C., Rienzi L., Navarro-Sánchez L., Cimadomo D., García-Pascual C.M., Albricci L., Soscia D., Valbuena D., Capalbo A., Ubaldi F. (2019). Embryonic Cell-Free DNA versus Trophectoderm Biopsy for Aneuploidy Testing: Concordance Rate and Clinical Implications. Fertil. Steril..

[21] Rubio C., Navarro-Sánchez L., García-Pascual C.M., Ocali O., Cimadomo D., Venier W., Barroso G., Kopcow L., Bahçeci M., Kulmann M.I.R. (2020). Multicenter Prospective Study of Concordance between Embryonic Cell-Free DNA and Trophectoderm Biopsies from 1301 Human Blastocysts. Am. J. Obstet. Gynecol..

[22] Shitara A., Takahashi K., Goto M., Takahashi H., Iwasawa T., Onodera Y., Makino K., Miura H., Shirasawa H., Sato W. (2021). Cell-Free DNA in Spent Culture Medium Effectively Reflects the Chromosomal Status of Embryos Following Culturing beyond Implantation Compared to Trophectoderm Biopsy. PLoS ONE.

[23] Vera-Rodriguez M., Diez-Juan A., Jimenez-Almazan J., Martinez S., Navarro R., Peinado V., Mercader A., Meseguer M., Blesa D., Moreno I. (2018). Origin and Composition of Cell-Free DNA in Spent Medium from Human Embryo Culture during Preimplantation Development. Hum. Reprod..

[24] Yeung Q.S.Y., Zhang Y.X., Chung J.P.W., Lui W.T., Kwok Y.K.Y., Gui B., Kong G.W.S., Cao Y., Li T.C., Choy K.W. (2019). A Prospective Study of Non-Invasive Preimplantation Genetic Testing for Aneuploidies (NiPGT-A) Using next-Generation Sequencing (NGS) on Spent Culture Media (SCM). J. Assist. Reprod. Genet..

[25] Fang R., Yang W., Zhao X., Xiong F., Guo C., Xiao J., Chen L., Song X., Wang H., Chen J. (2019). Chromosome Screening Using Culture Medium of Embryos Fertilised in Vitro: A Pilot Clinical Study. J. Transl. Med..

[26] Hanson B.M., Tao X., Hong K.H., Comito C.E., Pangasnan R., Seli E., Jalas C., Scott R.T. (2021). Noninvasive Preimplantation Genetic Testing for Aneuploidy Exhibits High Rates of Deoxyribonucleic Acid Amplification Failure and Poor Correlation with Results Obtained Using Trophectoderm Biopsy. Fertil. Steril..

[27] Li P., Song Z., Yao Y., Huang T., Mao R., Huang J., Ma Y., Dong X., Huang W., Huang J. (2018). Preimplantation Genetic Screening with Spent Culture Medium/Blastocoel Fluid for in Vitro Fertilization. Sci. Rep..

[28] Li X., Hao Y., Chen D., Ji D., Zhu W., Zhu X., Wei Z., Cao Y., Zhang Z., Zhou P. (2021). Non-Invasive Preimplantation Genetic Testing for Putative Mosaic Blastocysts: A Pilot Study. Hum. Reprod..

[29] Jiao J., Shi B., Sagnelli M., Yang D., Yao Y., Li W., Shao L., Lu S., Li D., Wang X. (2019). Minimally Invasive Preimplantation Genetic Testing Using Blastocyst Culture Medium. Hum. Reprod..

[30] Kuznyetsov V., Madjunkova S., Antes R., Abramov R., Motamedi G., Ibarrientos Z., Librach C. (2018). Evaluation of a Novel Non-Invasive Preimplantation Genetic Screening Approach. PLoS ONE.

[31] Kuznyetsov V., Madjunkova S., Abramov R., Antes R., Ibarrientos Z., Motamedi G., Zaman A., Kuznyetsova I., Librach C.L. (2020). Minimally Invasive Cell-Free Human Embryo Aneuploidy Testing (miPGT-A) Utilizing Combined Spent Embryo Culture Medium and Blastocoel Fluid –Towards Development of a Clinical Assay. Sci. Rep..

[32] Zhang J., Xia H., Chen H., Yao C., Feng L., Song X., Bai X. (2019). Less-Invasive Chromosome Screening of Embryos and Embryo Assessment by Genetic Studies of DNA in Embryo Culture Medium. J. Assist. Reprod. Genet..

[33] Sialakouma A., Karakasiliotis I., Ntala V., Nikolettos N., Asimakopoulos B. (2021). Embryonic Cell-Free DNA in Spent Culture Medium: A Non-Invasive Tool for Aneuploidy Screening of the Corresponding Embryos. In Vivo.

[34] Tšuiko O., Zhigalina D.I., Jatsenko T., Skryabin N.A., Kanbekova O.R., Artyukhova V.G., Svetlakov A.V., Teearu K., Trošin A., Salumets A. (2018). Karyotype of the Blastocoel Fluid Demonstrates Low Concordance with Both Trophectoderm and Inner Cell Mass. Fertil. Steril..

[35] Navarro-Sánchez L., García-Pascual C., Rubio C., Simón C. (2022). Non-Invasive Preimplantation Genetic Testing for Aneuploidies: An Update. Reprod. Biomed. Online.

[36] Cinnioglu C., Glessner H., Jordan A., Bunshaft S. (2023). A Systematic Review of Noninvasive Preimplantation Genetic Testing for Aneuploidy. Fertil. Steril..

[37] Chen Y., Gao Y., Jia J., Chang L., Liu P., Qiao J., Tang F., Wen L., Huang J. (2021). DNA Methylome Reveals Cellular Origin of Cell-Free DNA in Spent Medium of Human Preimplantation Embryos. J. Clin. Investig..

[38] Chow J.F.C., Lam K.K.W., Cheng H.H.Y., Lai S.F., Yeung W.S.B., Ng E.H.Y. (2024). Optimizing Non-Invasive Preimplantation Genetic Testing: Investigating Culture Conditions, Sample Collection, and IVF Treatment for Improved Non-Invasive PGT-A Results. J. Assist. Reprod. Genet..

[39] Handayani N., Aubry D., Boediono A., Wiweko B., Sirait B., Sini I., Polim A.A., Dwiranti A., Bowolaksono A. (2023). The Origin and Possible Mechanism of Embryonic Cell-Free DNA Release in Spent Embryo Culture Media: A Review. J. Assist. Reprod. Genet..

[40] Zhu D., Wang H., Wu W., Geng S., Zhong G., Li Y., Guo H., Long G., Ren Q., Luan Y. (2023). Circulating Cell-Free DNA Fragmentation Is a Stepwise and Conserved Process Linked to Apoptosis. BMC Biol..

[41] Rule K., Chosed R.J., Arthur Chang T., David Wininger J., Roudebush W.E. (2018). Relationship between Blastocoel Cell-Free DNA and Day-5 Blastocyst Morphology. J. Assist. Reprod. Genet..

[42] Practice Committees of the American Society for Reproductive Medicine and the Genetic Counseling Professional Group (2023). Clinical Management of Mosaic Results from Preimplantation Genetic Testing for Aneuploidy of Blastocysts: A Committee Opinion. Fertil. Steril..

[43] Capalbo A., Poli M., Rienzi L., Girardi L., Patassini C., Fabiani M., Cimadomo D., Benini F., Farcomeni A., Cuzzi J. (2021). Mosaic Human Preimplantation Embryos and Their Developmental Potential in a Prospective, Non-Selection Clinical Trial. Am. J. Hum. Genet..

[44] Treff N.R., Marin D. (2021). The “Mosaic” Embryo: Misconceptions and Misinterpretations in Preimplantation Genetic Testing for Aneuploidy. Fertil. Steril..

[45] Greco E., Greco P.F., Listorti I., Ronsini C., Cucinelli F., Biricik A., Viotti M., Meschino N., Spinella F. (2024). The Mosaic Embryo: What It Means for the Doctor and the Patient. Minerva Obstet. Gynecol..

