# Supplementary material for: Comparison of Non-Invasive and Minimally Invasive Preimplantation Genetic Testing for Aneuploidy Using Samples Derived from the Same Embryo Culture
Source: J Clin Med. 2024 Dec 25;14(1):33. doi: 10.3390/jcm14010033 (PMC11721003; doi:10.3390/jcm14010033)
Supplement: Supplementary file 1 [file jcm-14-00033-s001.zip › Table S1.pdf]

Table S1. Patients' and embryos' characteristics.

Characteristics	All patients N = 54 (56 IMSI cycles)	ni/miPGT-A optimization group N = 25 (25 IMSI cycles)	ni/miPGT-A group N = 30 (31 IMSI cycles)	*p*
Age of female partner [median (lower and upper quartile)]	36 (33-37)	36 (33-38)	36 (32-37)	0.540[a]
Age of male partner [median (lower and upper quartile)]	37 (35-40)	36 (34.5-40)	38 (35-40)	0,402[a]
Women ≥35 years old	35 (64.8%)	17 (68.0%)	19 (63.3%)	0.781[b]
Couples with severe male factor	11 (20.4%)	7 (28.0%)	4 (13.3%)	0.198[b]
Couples with previous unsuccessful IVF history	28 (51.9%)	14 (56.0%)	15 (50.0%)	0.788[b]
Couples with previous unsuccessful IUI history	24 (44.4%)	9 (36.0%)	15 (50.0%)	0.414[b]
Patients with aberrant karyotype	6 (11.1%)<br>46,XX,t(5;18)<br>46,XX,inv(6)(q16,2q21)<br>45,XX,rob(13;14)(q10;q10)<br>45,XX,rob(14;21)(q10;q10)<br>45,X[15]/46,XX[53]<br>47,XYY	1 (4.0%)<br>46,XX,inv(6)(q16,2q21)	5 (16.7%)<br>46,XX,t(5;18)<br>45,XX,rob(13;14)(q10;q10)<br>45,XX,rob(14;21)(q10;q10)<br>45,X[15]/46,XX[53]<br>47,XYY	0.205[b]
Number of MII oocytes fertilized by IMSI	461<br>(from 2 to 15/IMSI cycle)	202<br>(from 3 to 15/IMSI cycle)	259<br>(from 2 to 15/IMSI cycle)	
Number of viable embryos	276 (59.9%)<br>(from 1 to 15/IMSI cycle)	103 (51.0%)<br>(from 1 to 11/IMSI cycle)	173 (66.8%)<br>(from 1 to 15/IMSI cycle)	0.001[b]
Number of embryos with no development potential	185 (40.1%)<br>(from 0 to 9/IMSI cycle)	99 (49.0%)<br>(from 0 to 9/IMSI cycle)	86 (33.2%)<br>(from 0 to 8/IMSI cycle)	
Number of viable embryos subjected to PGT-A analysis	239<br>(from 1 to 15/IMSI cycle)	96<br>(from 1 to 8/IMSI cycle)	143<br>(from 1 to 15/IMSI cycle)	
**Embryos' class according to Gardner system**				
AA	146 (61.1%)	57 (59.4%)	89 (62.2%)	0.686[b] AA vs. AB&BB
AB	77 (32.2%)	38 (39.6%)	39 (27.3%)	
BB	16 (6.7%)	1 (1.0%)	15 (10.5%)	

a – *p* value of Mann-Whitney U test, b – *p* value of two-tailed Fisher's exact test. ni/miPGT-A – non-invasive/minimally invasive preimplantation genetic testing for aneuploidy, IMSI – intracytoplasmic morphologically selected sperm injection, IVF – in vitro fertilization, IUI – intrauterine insemination. The sum of couples in both groups is higher than the total number of couples included in the study because one couple who participated in 2 IMSI cycles was included in both groups.
