# Supplementary material for: Comparison of Non-Invasive and Minimally Invasive Preimplantation Genetic Testing for Aneuploidy Using Samples Derived from the Same Embryo Culture
Source: J Clin Med. 2024 Dec 25;14(1):33. doi: 10.3390/jcm14010033 (PMC11721003; doi:10.3390/jcm14010033)
Supplement: Supplementary file 1 [file jcm-14-00033-s001.zip › Table S2.pdf]

Table S2. Comparisons of genetic testing quality parameters and rates of concordance with TE between SCM samples derived from class AA embryos cultured for 5 and 6 days.

**Class AA embryos only**	**SCM (niPGT-A)**			
	**ni/miPGT-A group – day 5 N = 35**	**ni/miPGT-A group – day 6 N = 54**	***p***	**OR (95% CI)**
Samples with amplification failure	1 (2.9%)	0	0.393[a]	-
DNA concentration [ng/µl][b]	23.8 (21-29.1)	33.3 (31-36)	<0.001[c]	NA
NGS overall noise (DLR)[b]	0.33 (0.21-0.44)	0.25 (0.23-0.29)	0.097[c]	NA
Number of NGS reads after filtering[b]	275623 (150368-406809)	246407 (195590-318194)	0.438[c]	NA
Samples eligible for concordance analyses (with informative results of both niPGT-A and TE PGT-A)	N = 34	N = 53		
**Ploidy concordance of niPGT-A with PGT-A[d]**				
Concordant	26 (76.5%)	49 (92.5%)	0.054[a]	0.27 (0.07-0.96)
Discordant	8 (23.5%)	4 (7.6%)		
**Chromosome concordance of niPGT-A with PGT-A**				
Sex chromosomes	30 (88.2%)	50 (94.3%)	0.425[a]	-
Full (all chromosomes concordant)[e]	17 (50%)	42 (79.2%)	0.009[a]	0.26 (0.10-0.67)
Partial (at least one aberrant chromosome in agreement)	7 (20.6%)	6 (11.3%)	0.356[a]	-
None (no common aberrant chromosome between aneuploid samples)	2 (5.9%)	1 (1.9%)	0.558[a]	-
**Concordance/discordance characteristics**				
Euploid in niPGT-A - euploid in TE PGT-A (TN)	11 (32.4%)	31 (58.5%)	0.027[a]	0.34 (0.14-0.84)
Aneuploid in niPGT-A - aneuploid in TE PGT-A (TP)	15 (44.1%)	18 (34%)	0.372[a]	-
Euploid in niPGT-A - aneuploid in TE PGT-A (FN)	0	3 (5.7%)	0.278[a]	-
Aneuploid in niPGT-A - euploid in TE PGT-A (FP)	8 (23.5%)	1 (1.9%)	0.002[a]	16 (1.9-134.8)
Sensitivity (relative to TE PGT-A)	100%	85.7%		
Specificity (relative to TE PGT-A)	57.9%	96.9%		
Kappa statistics	0.548 moderate agreement	0.840 almost perfect agreement		

a – *p* value of two-tailed Fisher's exact test, b – median (lower and upper quartile), c – *p* value of Mann-Whitney U test. d – ploidy concordant results of niPGT-A (SCM) with results of PGT-A based on TE samples are defined as results euploid in both sample types or aneuploid in both sample types, ploidy discordant results include those euploid in PGT-A and aneuploid in niPGT-A, as well as aneuploid in PGT-A and euploid in niPGT-A, e – full chromosome concordance includes specimens with both results of niPGT-A and PGT-A euploid, or aneuploid for the same chromosomes. ni/miPGT-A – non-invasive/minimally invasive preimplantation genetic testing for aneuploidy, TE – trophectoderm, SCM – spent culture medium, NGS – next generation sequencing, DLR – derivative log ratio.
